# Human Adenovirus Serotype 3 Vector Packaged by a Rare Serotype 14 Hexon

**DOI:** 10.1371/journal.pone.0156984

**Published:** 2016-06-21

**Authors:** Xiaobo Su, Xingui Tian, Zaixue Jiang, Qiang Ma, Qian Liu, Xiaomei Lu, Rong Zhou

**Affiliations:** 1 Department of Medical Genetics and Cell Biology, School of Basic Science, Guangzhou Medical University, Guangzhou 511436, P.R. China; 2 State Key Laboratory of Respiratory Disease, Guangzhou Institute of Respiratory Disease, First Affiliated Hospital of Guangzhou Medical University, Guangzhou Medical University, Guangzhou 510120, P.R. China; 3 Dongguan Institute of Pediatrics, Dongguan Children's Hospital, Dongguan 523325, P.R. China; Swedish Neuroscience Institute, UNITED STATES

## Abstract

Recombinant adenovirus serotype 3 (rAd3), which infects cells through the receptor desmoglein 2 (DSG2), has been investigated as a vector for gene therapy or vaccination. However, pre-existing anti-vector immunity may limit the practical application of rAd3. In this study, we investigated the seroprevalence and neutralizing antibody (NAb) titers to Ad3 and alternate serotypes in normal healthy adults in southern China. Sera samples had a high seroprevalence (80.00%) against Ad3 and Ad7 (85.83%), compared with Ad14 (22.50%). Furthermore, 19.17% and 25.83% of samples had high-titer neutralizing antibodies to Ad3 and Ad7, respectively, compared with 3.33% against Ad14. We constructed a chimeric adenovirus, rAd3H14, designed to evade anti-vector immunity by replacing the enhanced green fluorescent protein (EGFP)-expressing hexon of the rAd3EGFP vector with a hexon from Ad14. The chimeric vector rAd3H14 was not neutralized *in vitro* efficiently by Ad3 NAbs using sera from mice and normal healthy human volunteers. Furthermore, in contrast to the unmodified vector rAd3EGFP, rAd3H14 induced robust antibody responses against EGFP in mice with high levels of pre-existing anti-Ad3 immunity. In conclusion, the chimeric vector rAd3H14 may be a useful alternative vector in adult populations with a high prevalence of Ad3 NAbs.

## Introduction

Adenovirus (Ad) vectors have been successfully used for vaccination and gene therapy against cancers and infectious diseases [1, 2]. However, the clinical applications of Ad-2- and Ad-5-based gene-transfer vectors, which currently are the most commonly used, are limited by two disadvantages: ‘pre-existing vector immunity’ in the majority of individuals and a lack of coxsackie and adenovirus receptor (CAR) expression in target cells [1–6].

Recently, several groups have developed vectors based entirely on species B including Ad3 vectors as candidates for vaccine design and gene transfer [5–13]. Unlike most Ad serotypes that utilize CAR as the primary attachment receptor [14, 15], the Ad3 of species B infects cells through the receptor desmoglein 2 (DSG2) [16, 17]. DSG2 is a calcium-binding transmembrane glycoprotein in the desmosomes of epithelial junctions, which is widely located in airway, gastrointestinal, and urinary tracts [18]. DSG2 is also present in nonepithelial tissues such as hematopoietic cells, dendritic cells, and cardiac muscle. Ad3-based vectors can potentially infect multiple cell types, which may be important for gene therapy targets with no or low-level expression of CAR [19]. More importantly, DSG2 was reported to be overexpressed in many epithelial cancers including squamous cell carcinomas, gastric cancer, breast cancer and bladder cancer, which justifies the application of the Ad3 vector for cancer therapy [16, 17]. Ad3 binding to DSG2 breaches epithelial barriers by transient intercellular junction opening, which may increase the therapeutic efficacy of anti-tumor drugs. Ad3-based vectors are relatively safe compared to Ad5-based vectors [20, 21]. Therefore, Ad3 vectors may be an alternative to Ad5-based vectors.

However, the clinical application of Ad vectors may be potentially limited by the high prevalence of pre-existing anti-vector immunity that decreases the expression of the transgene carried by the vector and thus affects the immunogenicity of the target antigens delivered. Both preclinical animal studies and clinical trials of Ad5-based vectors have demonstrated these limitations [22–24]. The high incidence of Ad3 infections in children might lead to a high prevalence of Ad3 neutralizing antibodies (NAb) in adult populations. However, there have been few reports on the seroprevalence of Ad3 and other members of species B in China [25].

The adenovirus capsid is an icosahedron comprising three structural proteins: the hexon, penton base, and fiber. It has been reported from our laboratory and others that the Ad3 and Ad5 hexon proteins are the major antigenic determinants recognized by serotype-specific NAbs [26–29]. Type-specific neutralizing epitopes of hexons have been proposed to reside within seven highly variable regions (HVRs) [30–32]. Our previous studies demonstrated that HVR1, 2, 4, 5, and 7 of Ad3 contain neutralizing epitopes [33]. Hexon modification [34–36] or exchange [30, 37] to construct modified Ad vectors is one of the approaches used to circumvent pre-existing anti-Ad immunity. In the present study, we investigated the seroprevalence of Ad3, Ad7 and Ad14 of species B in normal healthy adult individuals in southern China. We constructed a novel chimeric adenovirus rAd3H14 to circumvent anti-Ad3 immunity by replacing the hexon of Ad3 vector with the hexon from the rare serotype Ad14.

## Materials and Methods

### Ethics statement

Specific pathogen-free Balb/c mice were purchased from Guangdong Medical Laboratory Animal Center, and housed in the State Key Laboratory of Respiratory Disease with a barrier system. The mice were fed and maintained at 21±2°C, with 30–70% relative humidity and 12/12 hour light/dark cycle.The animal experiments were conducted in strict accordance with the recommendations of the Guide for the Care and Use of Laboratory Animals of the National Institutes of Health. All animals were housed individually and received humane care. During injection and sera sample collection, the mice were anesthetized with 1.5% isoflurane or 1 mL/kg weight 3% pentobarbital sodium to minimize their suffering. Mice were monitored each day and were humanely euthanized by the administration of pentobarbital sodium at the end of the study. All the mice represented healthy during the experiment, and there was no animal died prior to the experimental endpoint. Human sera samples were obtained with written informed consent in this study and were taken as part of the standard care. Experiments involving human samples and all animal procedures used in this work were evaluated and approved by the Ethic Committee of the First Affiliated Hospital of Guangzhou Medical University.

### Human serum samples

A total of 125 serum samples from healthy individuals were collected at random in 2014 by the Dongguan Institute of Pediatrics in Donguan and First Affiliated Hospital of Guangzhou Medical University in Guangzhou, southern China. Donors were aged from 20 to 49 years with a sex ratio of 1:1 without any other participant identifiers. The data were analyzed anonymously.

### Chimeric Ad vector construction

A recombinant adenovirus rAd3EGFP encoding the Ad3 GZ01 genome (GenBank accession no. DQ099432) and an enhanced green fluorescent protein (EGFP) were obtained as previously described [7]. Ad14 GZ01 strain (GenBank no. JQ824845.1) and Ad7 gz22 strain (GenBank no. KJ195466) were kindly provided by Professor Qiwei Zhang of Southern Medical University [38]. All the adenoviruses were cultured in HEp-2 cells or AD293 cells, and adenovirus particles were purified by standard CsCl gradient centrifugation as previously described [37]. The virus particle (VP) titers were determined by spectrophotometry using a conversion factor of 1.1×10^12^ VPs per absorbance unit at 260 nm [37].

The pBRAd3EGFP plasmid encoding the Ad-3 GZ-01 genome and EGFP with an E3 region deletion was constructed as previously described [7]. The shuttle vector pBRLR was constructed as previously described [26, 33]. In this study, the hexon-chimeric adenovirus rAd3H14 was obtained using the same strategy as previously described [26]. Briefly, the Ad14 hexon gene (H14) was obtained by PCR from Ad14 with the primers Hexon-F (5ʹ-CCG AGG CTG AGT TGC TTT CAA GAT GGC-3ʹ) and Hexon-R (5ʹ-GCA CTC TGA CCA CGT CGA AGA CTT CG-3ʹ), and then digested and ligated into pBRLR to generate the shuttle vector pBRLRH14. The resultant hexon protein was not different from the wild-type full-length H14. Then, the LR-H14 fragment was cloned into pBRAd3EGFP to generate the mutagenesis vector pBRAd3H14 using homologous recombinant technology in *Escherichia coli* strain BJ5183. The successful creation of these constructs was confirmed by restriction digestion and sequencing analyses. Finally, the chimeric adenovirus with Ad14 hexon replacement rAd3H14 was rescued in AD-293 cells. The full-length modified hexon genes of the viruses were identified by sequencing. The mutant virus was cultured with AD-293 cells in 20 100-mm dishes, then harvested and purified by standard CsCl gradient centrifugation as described above. The purified virions were analyzed by SDS-PAGE.

The growth curves and EGFP expression curves generated by the hexon-modified vector rAd3H14 were evaluated using previously described methods [26]. The purified vectors rAd3H14 and rAd3EGFP were seeded in serial dilutions to AD293 cells to determine the number of infectious particles (i.p.) by counting the cells with fluorescence at 48 hours after infection. Different amounts (10, 100 or 1000 VPs/cell) of the purified rAd3H14 vector were seeded to AD293 cells and cultured for 72 hours in 24-well plates. The infected cells were harvested and suspended in DMEM containing 2% fetal bovine serum, subjected to three freeze-thaw cycles, and centrifuged at 10,000 ×g for 30 min remove cell debris. The viral suspension was then diluted with DMEM containing 2% fetal bovine serum in a 10-fold dilution series to infect AD-293 cells cultured in 96-well plates. The number of i.p. was determined by counting the cells with fluorescence at 48 hours after infection, as previously described [39].

### Virus neutralization test

Female Balb/c mice aged 4–6 weeks were injected intramuscularly (i.m.) with 1×10^10^ VPs per mouse (about 10 μg total protein) of rAd3EGFP, Ad14 or rAd3H14, followed by two additional booster injections at 2-week intervals. Control mice were injected with phosphate buffered saline. At 2 weeks after the final immunization sera were collected, heat-inactivated and kept frozen for neutralization tests. Procedures for these animal experiments complied with all relevant federal guidelines and institutional policies. All animal studies were approved by our Institutional Animal Care and Use Committee.

The neutralizing antibodies against adenoviruses in human serum samples or mice sera were quantitatively detected by standard *in vitro* microneutralization tests. In brief, AD293 cells were seeded at a density of 2×10^4^ cells per well in 96-well plates and cultured for 24 hours. Concurrently, 100 TCID50 of the viruses were mixed with equal volumes of 2-fold serially diluted serum at 37°C for 1 hour. Then, the mixtures were adsorbed onto the corresponding cells and incubated for 72 hours. Titers from triplicate wells were read as the highest dilution of sera that inhibited virus growth without visible cytopathic effect.

### ELISA

To generate pre-existing Ad3 immunization, BALB/c mice were immunized by i.m. injection of 1×10^10^ VPs from w-Ad3 or phosphate buffered saline in a 0.1 ml volume per mouse, followed by a booster immunization with the same dose 3 weeks later. Three weeks later, the mice were subjected to an i.m. injection with 1×10^10^ VPs EGFP expressing vectors rAd3EGFP or rAd3H14 per mouse. Each group included eight mice. Two weeks after the final injection, serum antibody responses against EGFP were measured by indirect-ELISA with recombinant GFP protein as the capture antigen as described previously [26].

### Statistical analyses

Statistical significance was determined using Prism 5.0 software (GraphPad Prism Inc., La Jolla, CA, USA). Comparisons between two groups were made with Student’s t-test. Comparisons among groups were performed by one-way ANOVA with Bonferroni adjustments to account for multiple comparisons and *P* values of less than 0.05 were considered statistically significant.

## Results

### Seroprevalence and NAb titers to Ad3, Ad7 and Ad14

**Fig 1:**
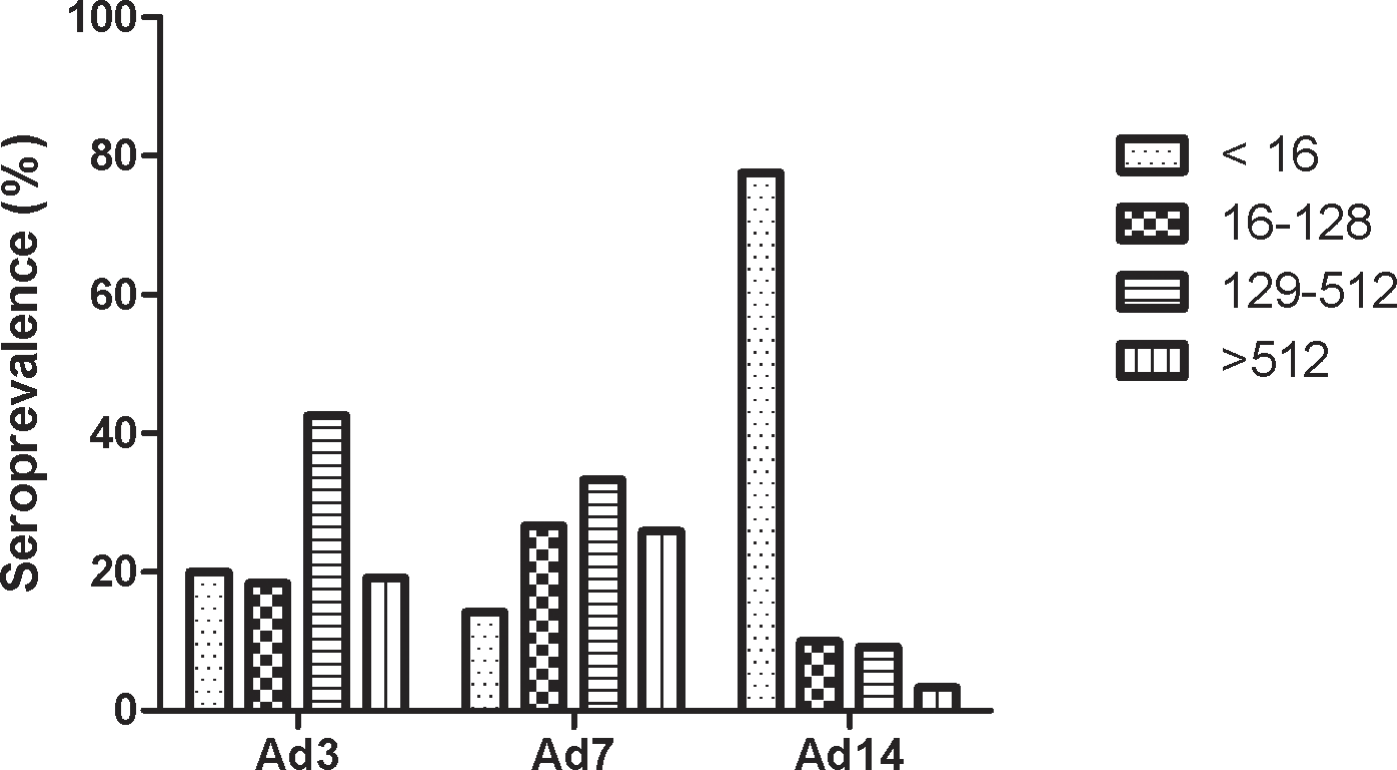
Seroprevalence and titer of NAbs against adenoviruses in 125 serum samples of healthy adults from southern China.

We initiated studies to assess the seroprevalence and NAb titers to Ad3 and alternate Ad serotypes in healthy adults. We collected serum samples from healthy donors from southern China and performed typical micro-neutralization assays. As shown in Fig 1, the Ad3 and Ad7 seroprevalences were 80% and 85.83%, respectively. In addition, 19.17% and 25.83% of the samples had notably high NAb titers (>512) against Ad3 and Ad7, respectively. In contrast, only 22.5% of the samples were positive for Ad14 NAbs, and fewer samples (3.33%) had high (>512) titers. These data demonstrated that Ad3-specific NAbs were nearly universal, and present at high titers in these populations in southern China. In contrast, Ad14 seroprevalence and titers were substantially lower. These results suggest that pre-existing anti-Ad3 immunity might substantially suppress the immunogenicity and clinical utility of rAd3 vector-based vaccines.

### Generation of a chimeric Ad3 vector with Ad14 hexon replacement

**Fig 2:**
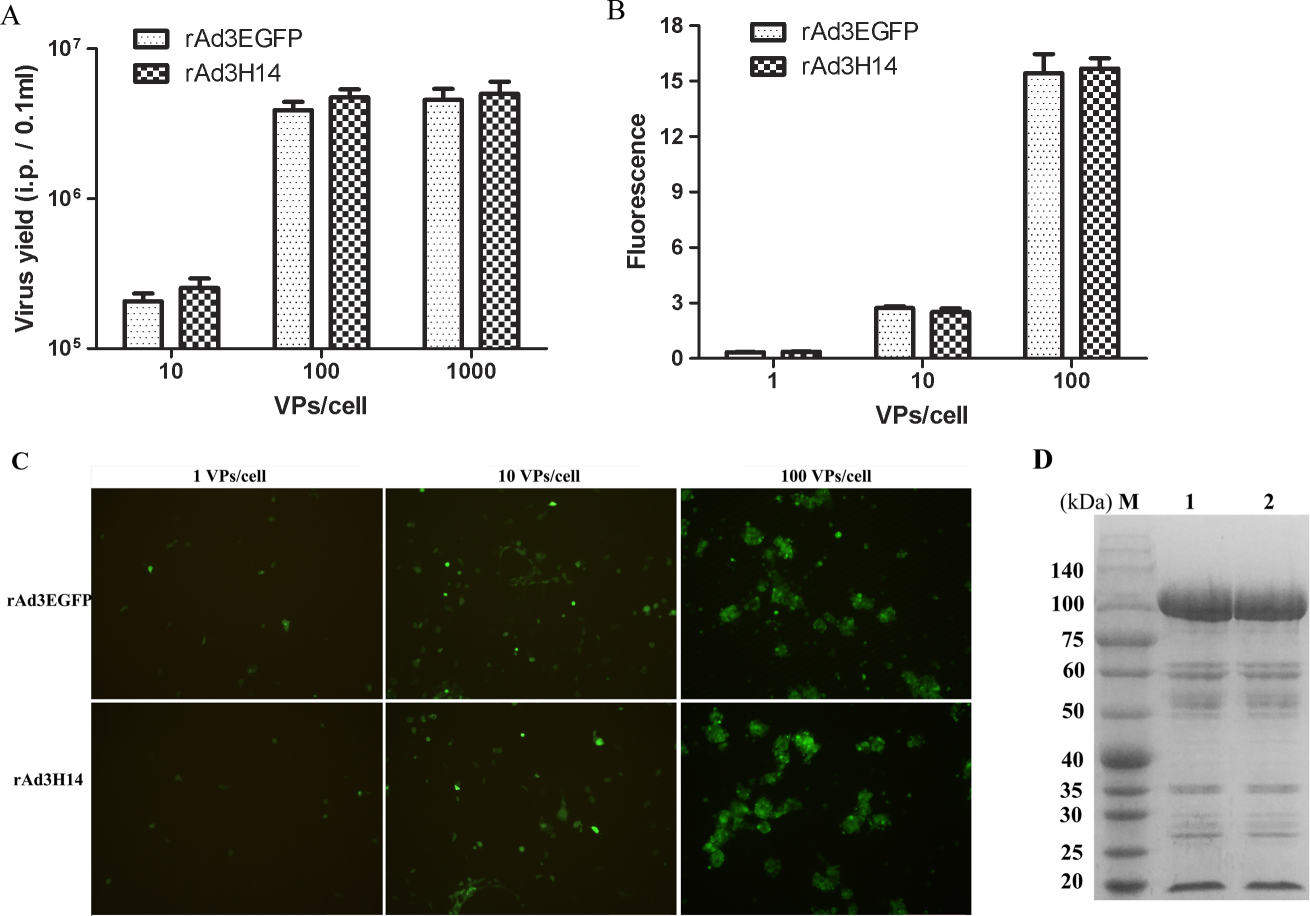
Construction of a hexon-chimeric rAd3H14 vector. (A) Different amounts (10, 100 or 1000 VPs/cell) of purified vectors rAd3H14 and rAd3EGFP were seeded to AD293 cells and cultured for 72 hours. Then the viruses were harvested and transfer to AD293 cells to determine the number of infectious particles (i.p.) by counting the cells with fluorescence at 48 hours after infection. (B) and (C) Transgene expression of rAd3H14 and rAd3EGFP vectors was measured by detecting the fluorescence of AD293 cells infected with 1, 10 or 100 VPs/cell of each vector. (D) Recombinant rAd3H14 confirmation by SDS-PAGE. M: marker (kDa). Lanes: 1: rAd3H14 and 2: rAd3EGFP virions. Each experiment was repeated independently at least three times. Data are shown as means ± SD.

The capsid of the chimeric rAd3H14 adenovirus with an H14 gene replacing the corresponding region of H3 in rAd3EGFP was successfully rescued and purified. The rAd3H14 vector was confirmed by PCR with Ad14-specific primers and by sequencing. The rAd3H14 vector grew efficiently to the same high yields as the parental rAd3EGFP vector (Fig 2A). We then examined the efficiency of gene transfer for rAd3H14 in AD293 cells with varying amounts of virus with the aid of an EGFP reporter. Transgene expression from rAd3H14 vectors expressing EGFP were comparable with that of the rAd3EGFP vector, suggesting that the gene transfer ability of rAd3H14 was not reduced by hexon replacement with H14 (Fig 2B and 2C). SDS-PAGE analysis demonstrated that the proteins complements of the purified chimeric rAd3H14 virion were equivalent to those of Ad3EGFP (Fig 2D). The purified chimeric viruses and Ad3EGFP had similar VP:i.p. ratios of about 100:1 to 150:1. Two additional individual viral preparations generated similar results.

### Chimeric rAd3H14 vector is not neutralized efficiently by Ad3 NAbs

**Fig 3:**
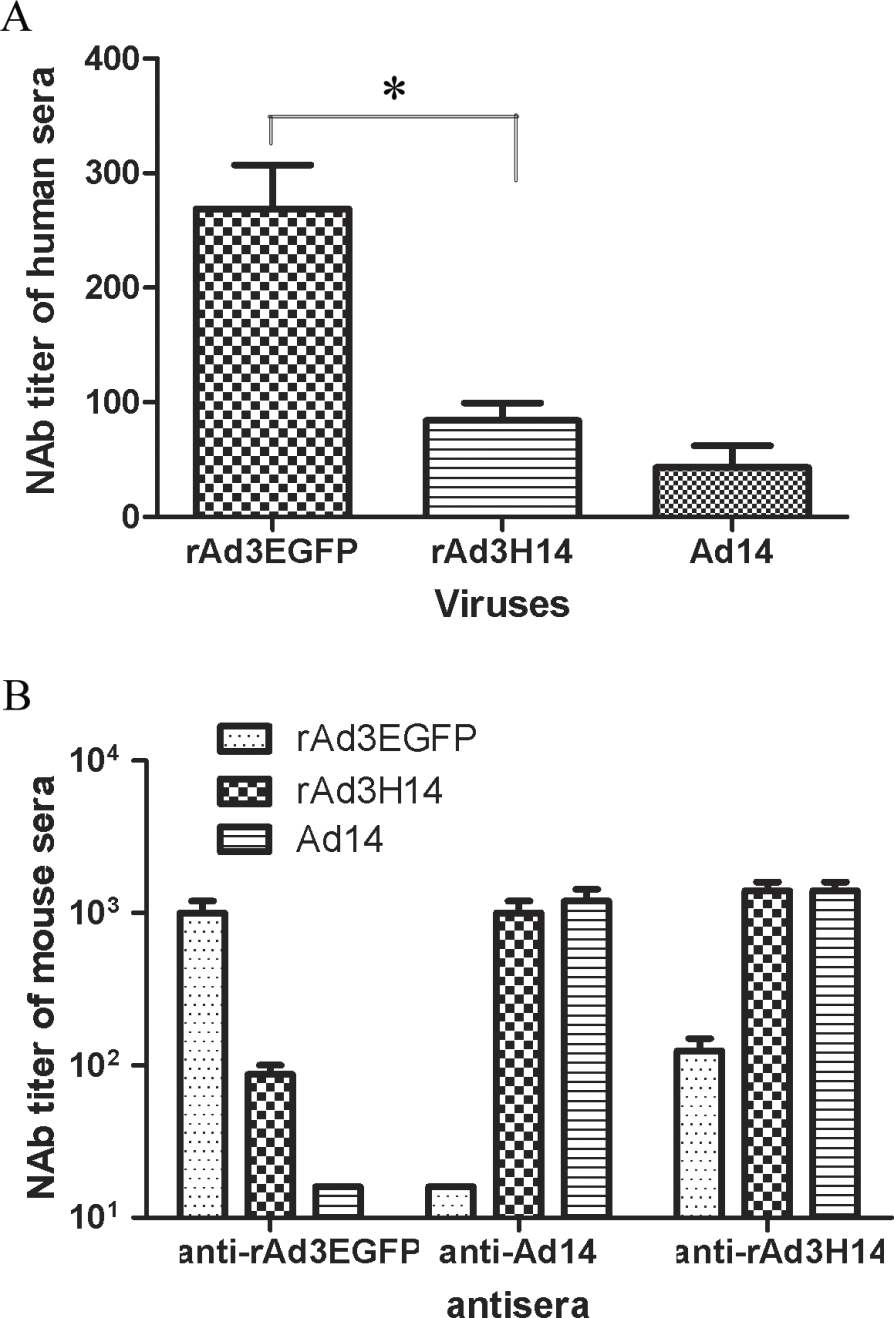
Chimeric rAd3H14 vector evades the neutralization of anti-Ad3 immunity. Forty serum samples from healthy adults (A) or serum from rAd3EGFP-, rAd3H14- or Ad14-immunized mice (n = 5 per group) (B) were assessed for NAb titers to rAd3EGFP, rAd3H14 or Ad14 viruses. Each experiment was repeated independently at least three times. Data are shown as means ± SEM. * *P*<0.05.

Our previous study suggested that the majority of Ad3-specific NAb activity in mice was directed against the Ad3 hexon [26]. In this study we assessed vector-specific NAb titers in 40 human serum samples of healthy individuals from southern China (Fig 3A). The median rAd3EGFP-specific NAb titer was 269, whereas the median NAb titer against rAd3H14 was significantly lower at 84 (*P*<0.05, Dunn's Multiple Comparison Test). The NAb titers of six typical samples are shown in Table 1. These data confirm Ad3-specific NAbs in humans are principally directed against the hexon. The residual low-titer NAbs against rAd3H14 presumably represent Ad3 fiber- or penton-specific NAbs, because Ad14-specific NAbs were particularly lower in these samples. Overall, the replacement of H3 with H14 significantly reduced the NAb titers against vectors in healthy human adults.

**Table 1:** NAb titers against chimeric rAd3H14 and its parental rAd3EGFP and Ad14 from human serum samples.

Adenovirus	Human serum sample					
	2#	24#	22#	5#	36#	40#
Ad14	0^a^	0^a^	0^a^	128	512	256
rAd3EGFP	1024	512	64	512	128	0^a^
rAd3H14	256	64	0^a^	128	512	256

^a^ No detectable neutralization.

#: the serum serial number

We next confirmed these findings by *in vitro* neutralization tests using serum from experimentally immunized mice. As shown in Fig 3B, serum from rAd3EGFP-immunized mice exhibited 8–16 fold lower NAb titers to rAd3H14, consistent with the results obtained with the human samples. Conversely, serum from rAd3H14-immunized mice had comparable NAb titers to Ad14 and rAd3H14, but exhibited 8–16 fold lower NAb titers to rAd3EGFP. As expected, there was no cross-neutralization between rAd3EGFP and Ad14. These data clearly demonstrated that Ad3- and Ad14-specific NAbs were principally directed against the hexons, although Ad3 fiber-specific NAbs were also present but at much lower titers than Ad3 hexon-specific NAbs. These results obtained from human and mouse serum suggested that rAd3H14 vectors effectively circumvented anti-Ad3 immunity *in vitro*.

### Chimeric rAd3H14 vector induces transgene-specific humoral immune responses in mice with pre-existing immunity against Ad3

**Fig 4:**
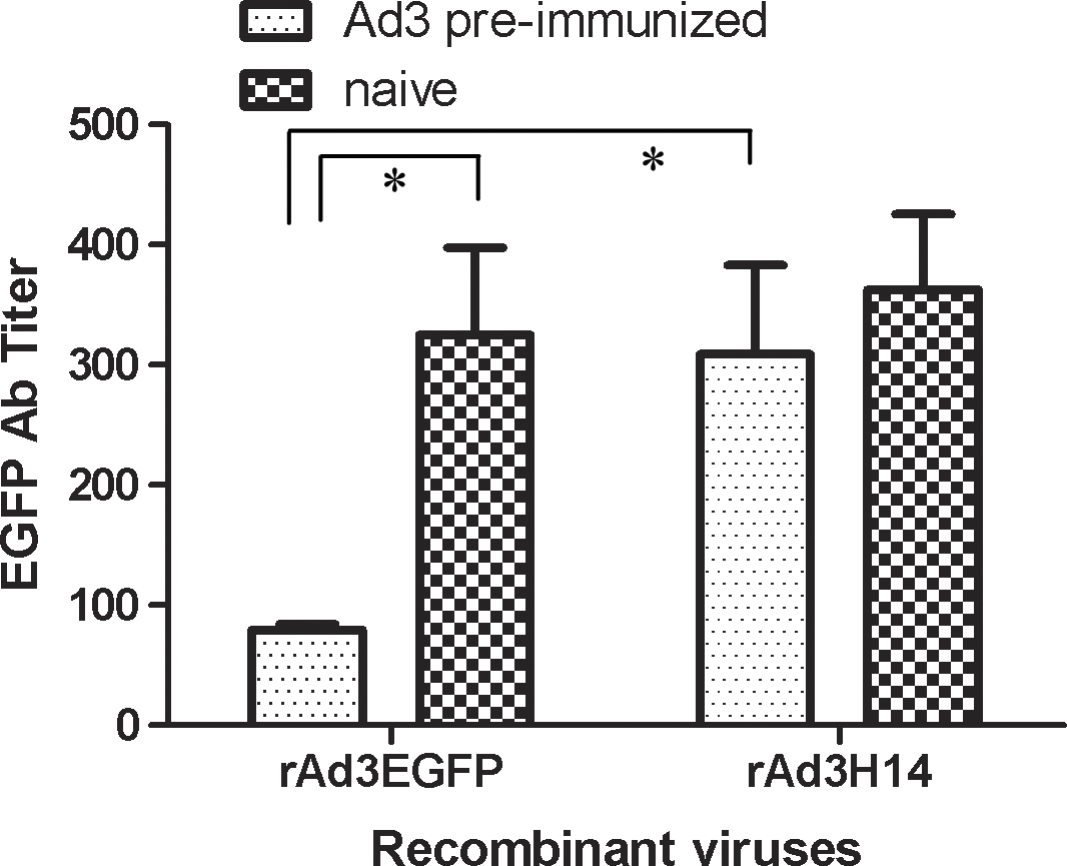
Immunogenicity of the hexon chimeric rAd3H14 vector expressing EGFP in mice with anti-Ad3 immunity. Naive mice or mice with anti-Ad3 immunity were immunized intramuscularly with 10^10^ VPs of rAd3EGFP or rAd3H14 vector. EGFP-specific humoral immune responses were detected by indirect-ELISA with recombinant GFP protein as the capture antigen at 2 weeks after immunization. Each experiment was repeated independently at least three times. Data are shown as means ± SEM. * *P*<0.05.

We then assessed the immunogenicity of the chimeric rAd3H14 vector and rAd3EGFP expressing EGFP in mice either with or without anti-Ad3 immunity. Groups of mice were preimmunized twice with 10^10^ VPs of wild-Ad3 to generate anti-Ad3 immunity. These mice had Ad3-specific NAb titers of 1,024–2,048. Mice with anti-Ad3 immunity and naive mice (n = 8 per group) were immunized i.m. with a single injection of 10^10^ VPs of rAd3EGFP or rAd3H14 vectors. As shown in Fig 4, in naive mice, rAd3H14 showed a similar immunogenicity with rAd3EGFP. In mice with anti-Ad3 immunity, the immunogenicity of rAd3EGFP was significantly suppressed (*P*<0.01, one-way ANOVA with Bonferroni adjustments to account for multiple comparisons). However, the immunogenicity of rAd3H14 remained unaffected by anti-Ad3 immunity. In fact, responses elicited by rAd3H14 were significantly higher than those elicited by rAd3EGFP in mice with anti-Ad3 immunity (*P*<0.01, one-way ANOVA with Bonferroni adjustments to account for multiple comparisons). These data reflect the capacity of this chimeric vector to circumvent anti-Ad3 immunity.

## Discussion

In this study, we investigated the seroprevalence and neutralizing antibody titers of Ad3, Ad7, and Ad14 of species B in normal healthy adult individuals from southern China. A chimeric rAd3H14 adenovirus was constructed to circumvent anti-Ad3 immunity by replacing the hexon of the Ad3 vector with the hexon from the rare serotype Ad14.

This is the first report of the seroprevalence of Ad3, Ad7 and Ad14 in China. It was demonstrated that Ad3- and Ad7-specific NAbs were nearly universal and present at high titers in adult populations in southern China. In contrast, the Ad14 seroprevalence and titers were substantially lower (Fig 1). The prevalences are in accord with the low incidence of Ad14 infections in China, and the high incidence of Ad3 infections, especially in children. However, the low proportion of samples with NAbs against Ad14 suggests that there may be little protection in the population of southern China and indicates that this area should be frequently surveyed for Ad14 infection. This report also provided important foundational data for developing novel Ad vectors.

Adenovirus serotype 3 vectors have been developed as candidates for vaccine design and gene transfer [7–9]. However, pre-existing anti-vector immunity is a major obstacle to the clinical application of adenovirus vectors [22–24]. Therefore, it is necessary to develop modified Ad3 vectors that can evade anti-vector immunity. Hexon switching or modification to construct modified Ad5 vectors has been successfully used to circumvent pre-existing anti-Ad5 immunity [34–36]. We previously constructed a chimeric adenovirus rAd3/H7 that evaded anti-Ad3 neutralization by replacing the hexon of the Ad3 vector with the Ad7 hexon [26]. However, rAd3/H7 did not evade anti-vector immunity because the Ad7-specific NAbs in human populations were universal and had a similar high titer as for Ad3 (Fig 1). Therefore, we constructed a chimeric rAd3H14 adenovirus by replacing the hexon of rAd3EGFP with the hexon from Ad14. The rAd3H14 vector was produced at a high titer similar to that of the parental rAd3EGFP vector (Fig 2A and 2D). Furthermore, the transgene expressing ability of rAd3H14 was not reduced (Fig 2B). This result demonstrates that hexon exchange among adenoviruses from the same species without affecting the packaging and gene transfer efficiency is feasible [26, 40, 41]. These data are important for rAd3H14 as a substitute transgene vector of rAd3EGFP.

The *in vitro* neutralization study with rAd3H14 suggested that the majority of Ad3-specific NAb activity in mice was directed against the Ad3 hexon (Fig 3). This conclusion is in accord with our previous study using rAd3/H7[26]. However, of note, here we demonstrated that Ad3- and Ad14-specific NAb activities were primarily directed against the hexon in human sera, although the fiber or penton base of Ad3 also contributed to the production of NAbs (Table 1).

As shown in Fig 3A and 3B, the chimeric rAd3H14 vector was not neutralized efficiently *in vitro* by Ad3 NAbs in human sera or immunized mice sera. More importantly, the median NAb titer in 40 human sera samples against rAd3H14 was significantly lower than that against rAd3EGFP (Fig 3A), which demonstrated that rAd3H14 circumvented anti-vector neutralization in most individuals. We also demonstrated transgene-specific humoral immune responses in mice with pre-existing immunity against Ad3 and confirmed that IgG antibody titers elicited by rAd3H14 were significantly higher than those elicited by rAd3EGFP (Fig 4). These data reflect the potentiality of the chimeric rAd3H14 vector to circumvent anti-vector immunity.
